# Trends in overweight and obesity among reproductive-age women in Bangladesh: Analysis of nationally representative surveys over a decade

**DOI:** 10.1371/journal.pone.0347419

**Published:** 2026-04-15

**Authors:** Urby Saraf Anika, Md. Abdur Rafi, Md. Shajedur Rahman Shawon, Md. Golam Hossain

**Affiliations:** 1 Department of Public Health and Informatics, Bangladesh Medical University, Dhaka, Bangladesh; 2 Epidemiology and Population Health Division, FibyLab, Dhaka, Bangladesh; 3 Centre for Big Data Research in Health, UNSW Sydney, Sydney, New South Wales, Australia; 4 Department of Statistics, University of Rajshahi, Rajshahi, Bangladesh; Austin College, UNITED STATES OF AMERICA

## Abstract

**Background:**

Overweight and obesity are emerging public health concerns in Bangladesh, contributing to the rising burden of non-communicable diseases. We aimed to examine trends in prevalence of overweight and obesity among reproductive-age women in Bangladesh over the past decade and assess socioeconomic inequalities in their distribution.

**Methods:**

We analyzed nationally representative data from four rounds of the Bangladesh Demographic and Health Survey (BDHS) conducted in 2011, 2014, 2017–18, and 2022, including a total of 60,921 women aged 15–49 years. Body mass index (BMI) was classified using Asian-specific cut-offs. Prevalence estimates of overweight and obesity were calculated for each survey year and stratified by age, residence, educational attainment, and wealth quintile. Log-linear regression was used to estimate annual percentage changes (APC) in overweight and obesity. Socioeconomic inequality was quantified using concentration curves and Erreygers-corrected concentration indices (CIX).

**Results:**

Overall prevalence of overweight and obesity increased from 13.1% in 2011 to 18.7% in 2022 (APC 3.1%), and 17.7% to 36.6% (APC 6.8%), respectively. Rural women experienced faster relative increases in both overweight (APCs 4.4% vs 1.1%).and obesity (APCs 9.2% vs 4.3%) compared with urban women. Women in the poorest and poorer wealth quintiles showed the largest APCs for obesity (14.2% and 14.1%, respectively). The CIX for overweight declined from 0.098 to 0.031, and for obesity from 0.200 to 0.141, indicating a modest reduction in inequality concentrated among wealthier groups over time.

**Conclusions:**

Overweight and obesity are increasing rapidly among reproductive-age women of Bangladesh, with faster rises among rural and lower-income groups. Policies and interventions should address both overall prevalence and shifting socioeconomic patterns to reduce the future burden of non-communicable diseases.

## Background

Bangladesh, like many low- and middle-income countries (LMICs), is experiencing a rapid epidemiological transition, with non-communicable diseases (NCDs) emerging as the leading contributors to morbidity and mortality [1–3]. Once dominated by infectious diseases and undernutrition, the country is now confronted with a growing burden of NCDs, which represents 14 of the top 20 causes of death [1]. Obesity is recognized as one of the most important modifiable risk factors for NCDs including type 2 diabetes, hypertension, cardiovascular disease, and certain cancers [4].

Globally, obesity has been rising at an alarming pace [5]. While obesity has traditionally been perceived as a problem of high-income countries, LMICs like Bangladesh are witnessing an equally rapid increase [6]. In Bangladesh, the prevalence of overweight and obesity has increased from 17% to 49% in women and 21% to 34% in men in the last two decades and studies have consistently shown this higher prevalence of obesity among women compared to men, particularly in urban areas and among those belonging to higher socioeconomic strata [7]. This higher prevalence of obesity in women of reproductive age is not only associated with increased health risk for the women themselves but also for maternal, fetal, and child health outcomes. Overweight and obesity increase the risk of adverse pregnancy outcomes, including gestational diabetes, pre-eclampsia, stillbirth, and cesarean delivery [8,9]. Furthermore, maternal obesity has been linked to long-term health risks for offspring, such as childhood obesity and metabolic disorders [10]. Hence, this group of population represent a particularly important demographic for obesity research.

Several studies reported a rising trend in prevalence of overweight and obesity among women in Bangladesh [7,11,12]. Besides, previous studies also reported significant regional, urban-rural, and socioeconomic differences in these burdens, with higher prevalence consistently observed among urban residents, women with higher educational attainment, and those from wealthier households [13–16]. However, majority of these studies relied on pooled estimates to compare population subgroups, with limited exploration in the temporal changes in these differences. It remains unclear whether the increase in obesity has occurred uniformly across socioeconomic strata or whether differences have widened or narrowed over time. Furthermore, earlier studies did not explicitly evaluate differences in the rate of increase between groups, an approach that is essential to understand the dynamics of the obesity epidemic and to identify populations experiencing the most rapid rise in prevalence. Against this backdrop, in the present study, we aimed to examine trends in overweight and obesity among reproductive-age women in Bangladesh across wealth quintiles, education levels, place of residence, and other sociodemographic characteristics using nationally representative data.

## Methods

### Data source

We used data from four consecutive rounds of the Bangladesh Demographic and Health Survey (BDHS), conducted in 2011, 2014, 2017−18, and 2022 [17–20]. Each BDHS was a nationally representative household survey implemented by the National Institute of Population Research and Training (NIPORT) in collaboration with ICF International. The surveys followed a two-stage stratified cluster sampling design. In the first stage, enumeration areas were randomly selected from the most recent census, stratified by urban and rural residence within each administrative division. In the second stage, households were randomly sampled from these clusters, and all ever-married women aged 15–49 years who were usual residents or stayed in the household the previous night were eligible to participate. For this study, we pooled all women with valid anthropometric data from the four survey waves. Pregnant women were excluded from our analysis to avoid distortion of BMI distributions. Each BDHS provided sample weights to account for unequal selection probabilities and nonresponse, and we applied these weights in all analyses. We also adjusted for the complex survey design by incorporating clustering and stratification.

### Variables

#### Outcome variable.

Our outcome variable was body mass index (BMI), calculated as weight in kilograms divided by height in meters squared. In each round of BDHS, anthropometric measurements were collected by trained interviewers using standardized protocols. Height was measured to the nearest 0.1 cm with a portable stadiometer, and weight to the nearest 0.1 kg with calibrated digital scales. For primary analysis, we classified BMI using Asian-specific cut-offs, categorized as underweight (<18.5 kg/m²), normal weight (18.5–22.9 kg/m²), overweight (23.0–27.4 kg/m²), or obese (≥27.5 kg/m²) [21]. For secondary analysis, we classified BMI using WHO cut-offs, categorized as underweight (<18.5 kg/m²), normal weight (18.5–24.9 kg/m²), overweight (25.0–29.9 kg/m²), or obese (≥30.0 kg/m²) [22]. For the purposes of trend analyses, normal weight served as the reference group.

#### Independent variables.

The main independent variable was survey year. We also examined sociodemographic characteristics, such as age (grouped as 15–29, 30–39, and 40–49 years), place of residence (urban or rural), educational attainment (categorized as no education, primary, secondary, or higher, based on the highest level completed), and household wealth quintile. Household wealth was measured using the DHS wealth index, which is derived from principal component analysis of household assets and dwelling characteristics, and divided into quintiles (poorest to richest) [17–20].

### Ethical considerations

The BDHS protocols were reviewed and approved by the Bangladesh Medical Research Council and the ICF Institutional Review Board. Informed consent was obtained from all participants at the time of data collection. As this analysis used secondary, de-identified data, no additional ethical approval was required.

### Statistical analysis

We restricted analyses to women with complete and valid information on height, weight, age, education, residence, and household wealth. Women who were pregnant at the time of the survey or had missing anthropometric data were excluded. Because the proportion of missing data was small and assumed to be missing at random, we performed complete-case analysis without imputation.

We first estimated the prevalence of overweight, and obesity separately for each survey round, both overall and stratified by age group, residence, education, and wealth quintile. Prevalence was expressed as the weighted proportion of women in each BMI category, and 95% confidence intervals (CI) were calculated for all estimates.

To assess temporal trend, we calculated absolute changes in prevalence between the first and last survey years (2011 and 2022). Absolute change was defined as the difference in prevalence across these two time points, with 95% CIs obtained by combining the standard errors of the individual estimates. Then, we modeled trends using log-linear regression, with log-transformed prevalence as the dependent variable and survey year as the independent variable. This approach provided an estimate of the average yearly percentage change in prevalence, summarized as the annual percentage change (APC). We reported APC values with 95% CIs and p-values to assess the strength and direction of trends. To evaluate whether trends differed across subgroups, we extended the log-linear models by including interaction terms between survey year and each sociodemographic variable. Likelihood ratio tests compared models with and without the interaction term, and a p-value less than 0.05 was considered evidence of a differential trend.

To assess socioeconomic inequalities in overweight and obesity, we constructed concentration curves (CCs) and concentration indices (CIXs). The CC visually represents the cumulative proportion of women, ranked by household wealth, against the cumulative proportion of women classified as overweight or obese. A CC below the line of equality indicates that obesity is more concentrated among women from wealthier households, whereas a curve above the equality line would indicate higher concentration among women from poorer households. Because obesity is a binary outcome, we applied Erreygers’ correction to the CIX to ensure an accurate and normalized measure of socioeconomic inequality. Positive Erreygers CIX values indicate that obesity is concentrated among women in higher wealth quintiles, while negative values indicate concentration among women in lower wealth quintiles.

We conducted all statistical analyses in R version 4.4.2. We used the survey package to apply sampling weights and account for clustering and stratification.

## Results

### Sociodemographic characteristics

We included a total of 60,921 women of reproductive age in the analysis across the four BDHS rounds. The proportion of younger women (15–29 years) declined from 47% in 2011 to 39% in 2022, with corresponding increases in the 30–39 and 40–49 age groups. Approximately, one-third of participants lived in urban areas throughout the period. Educational attainment improved, with women reporting no formal education decreasing from 27% to 14% and those with secondary or higher education rising from 43% to 59% (Table 1).

**Table 1:** Sociodemographic characteristics of the participants (n = 60,921).

Characteristics	BDHS 2011, n = 16,271	BDHS 2014, n = 16,622	BDHS 2017−18, n = 18,680	BDHS 2022, N = 9,348
Age (years)				
15-29	7,634 (46.92)	7,511 (45.19)	7,944 (42.53)	3,660 (39.15)
30-39	4,737 (29.11)	5,180 (31.16)	6,147 (32.91)	3,291 (35.21)
40-49	3,900 (23.97)	3,931 (23.65)	4,589 (24.57)	2,397 (25.64)
Residence				
Urban	5,708 (35.08)	5,763 (34.67)	6,797 (36.39)	3,281 (35.10)
Rural	10,563 (64.92)	10,859 (65.33)	11,883 (63.61)	6,067 (64.90)
Educational attainment				
No formal education	4,385 (26.95)	4,039 (24.30)	3,111 (16.65)	1,322 (14.14)
Primary	4,857 (29.85)	4,874 (29.32)	5,965 (31.93)	2,464 (26.36)
Secondary	5,731 (35.22)	6,152 (37.01)	7,115 (38.09)	4,202 (44.95)
Higher	1,298 (7.98)	1,557 (9.37)	2,489 (13.32)	1,360 (14.55)
Wealth quintile				
Poorest	2,811 (17.28)	2,999 (18.04)	3,554 (19.03)	1,703 (18.22)
Poorer	2,995 (18.41)	3,102 (18.66)	3,574 (19.13)	1,766 (18.89)
Middle	3,128 (19.22)	3,381 (20.34)	3,639 (19.48)	1,893 (20.25)
Richer	3,472 (21.34)	3,534 (21.26)	3,808 (20.39)	1,948 (20.84)
Richest	3,865 (23.75)	3,606 (21.69)	4,105 (21.98)	2,038 (21.80)

### Trend in overweight

**Fig 1:**
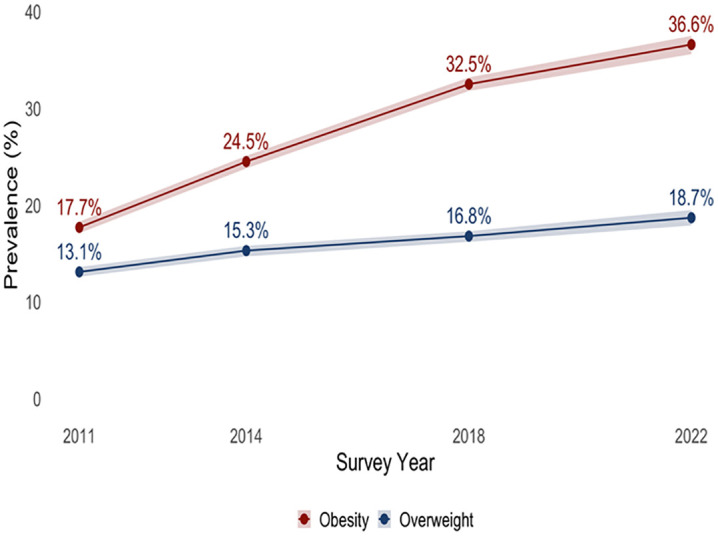
Trend in prevalence of overweight and obesity among reproductive-age women according to Asian BMI cut-off.

Overall, prevalence of overweight among reproductive-age women increased steadily from 13.1% in 2011 to 18.7% in 2022 according to the Asian cut-off, corresponding to an APC of 3.1% (Fig 1).

Similarly, prevalence of overweight according to WHO cut-off among reproductive-age women increased from 14.5% in 2011 to 28.5% in 2022, corresponding to an APC of 6.2% (S1 Table).

Increases were observed across all age groups, with the most rapid rise among women aged 15–29 years (APC 3.8%) and slightly slower increases among women aged 30–39 and 40–49 years (APCs 2.1% and 2.9%, respectively), with no statistically significant differences across age groups (p = 0.285). The pace of increase differed by place of residence, with rural women experiencing a more rapid rise than their urban counterparts (APCs 4.4% vs 1.1%; p = 0.014). Educational attainment also influenced trends, with the steepest increase among women with higher education (APC 5.8%) and a more modest rise among those with no formal education (APC 2.7%). Women from the poorest and poorer quintiles showed the largest APCs (8.7% and 5.5%, respectively), whereas the richest quintile showed minimal change (APC 0.2%; p = 0.011) (Table 2). These gradients persist when the BMI was categorized according to the WHO cut-off, except for the wealth quintiles (S1 Table). Prevalence of overweight increased across all geographic regions, with the highest APC in Sylhet and Rangpur divisions (6.0% and 4.9%, respectively). The urban-rural difference was highest in Sylhet division, followed by Dhaka, Barisal, and Rangpur divisions (S2 Table).

**Table 2:** Trends in overweight and obesity among the participants (n = 60,921).

Characteristics	Prevalence (%)				Absolute change, % (95% CI)	APC, % (95% CI)	p-value*	p-value**
	BDHS 2011	BDHS 2014	BDHS 2017−18	BDHS 2022				
**Overweight**								
Overall	13.1 (12.6 - 13.6)	15.3 (14.7 - 15.8)	16.8 (16.2 - 17.3)	18.7 (17.9 - 19.5)	5.56 (4.62 - 6.51)	3.13 (2.38 - 3.89)	<0.001	
Age (years)								0.285
15-29	11.3 (10.6 - 12.0)	13.7 (13.0 - 14.5)	16.6 (15.8 - 17.4)	17.0 (15.8 - 18.2)	5.69 (4.28 - 7.10)	3.82 (1.94 - 5.75)	<0.001	
30-39	15.4 (14.3 - 16.4)	17.0 (16.0 - 18.0)	17.0 (16.1 - 18.0)	19.9 (18.5 - 21.3)	4.53 (2.83 - 6.24)	2.12 (1.05 - 3.20)	<0.001	
40-49	14.0 (12.9 - 15.1)	15.9 (14.8 - 17.0)	16.8 (15.7 - 17.8)	19.6 (18.1 - 21.2)	5.62 (3.70 - 7.55)	2.90 (2.11 - 3.71)	<0.001	
Residence								0.014
Urban	16.1 (15.2 - 17.1)	16.8 (15.8 - 17.7)	16.9 (16.0 - 17.8)	18.5 (17.2 - 19.8)	2.37 (0.73 - 4.00)	1.14 (0.52 - 1.75)	<0.001	
Rural	11.5 (10.9 - 12.1)	14.5 (13.8 - 15.1)	16.7 (16.1 - 17.4)	18.8 (17.8 - 19.8)	7.29 (6.13 - 8.44)	4.40 (2.98 - 5.84)	<0.001	
Educational attainment								0.089
No formal education	10.8 (10.0 - 11.6)	12.6 (11.7 - 13.6)	13.2 (12.3 - 14.1)	15.4 (14.3 - 16.6)	4.59 (3.30 - 5.88)	2.66 (1.57 - 3.76)	<0.001	
Primary	13.1 (12.2 - 13.9)	15.4 (14.6 - 16.3)	16.3 (15.4 - 17.2)	18.4 (17.3 - 19.5)	5.29 (4.04 - 6.54)	2.95 (1.68 - 4.26)	<0.001	
Secondary	17.1 (16.1 - 18.1)	19.2 (18.2 - 20.2)	21.4 (20.4 - 22.4)	22.4 (21.2 - 23.5)	5.36 (4.13 - 6.59)	3.36 (1.77 - 4.99)	<0.001	
Higher	15.3 (14.0 - 16.6)	18.8 (17.7 - 19.9)	22.3 (21.2 - 23.4)	24.3 (22.9 - 25.7)	9.01 (6.91 - 11.10)	5.82 (4.09 - 7.59)	<0.001	
Wealth quintile								0.011
Poorest	6.0 (5.2 - 6.9)	10.1 (9.0 - 11.2)	14.7 (13.5 - 15.8)	15.4 (13.7 - 17.1)	9.34 (7.41 - 11.26)	8.74 (3.85 - 13.87)	<0.001	
Poorer	10.1 (9.0 - 11.2)	13.5 (12.3 - 14.7)	15.3 (14.2 - 16.5)	18.7 (16.9 - 20.6)	8.66 (6.54 - 10.78)	5.45 (3.77 - 7.15)	<0.001	
Middle	13.0 (11.8 - 14.2)	15.4 (14.1 - 16.6)	18.6 (17.3 - 19.8)	19.0 (17.2 - 20.8)	6.04 (3.91 - 8.16)	3.62 (1.96 - 5.31)	<0.001	
Richer	15.3 (14.1 - 16.5)	18.5 (17.2 - 19.8)	17.8 (16.6 - 19.0)	20.6 (18.8 - 22.4)	5.31 (3.15 - 7.47)	2.29 (0.56 - 4.04)	0.009	
Richest	18.8 (17.6 - 20.0)	17.9 (16.6 - 19.1)	17.3 (16.2 - 18.5)	19.2 (17.5 - 20.9)	0.45 (−1.66 - 2.56)	0.16 (−1.17 - 1.50)	0.820	
**Obesity**								
Overall	17.7 (17.1 - 18.3)	24.5 (23.8 - 25.1)	32.5 (31.8 - 33.2)	36.6 (35.6 - 37.5)	18.86 (17.72 - 20)	6.77 (4.35 - 9.25)	<0.001	
Age (years)								0.812
15-29	12.5 (11.8 - 13.3)	18.0 (17.1 - 18.8)	24.2 (23.2 - 25.1)	26.8 (25.4 - 28.3)	14.32 (12.70 - 15.94)	7.12 (4.22 - 10.09)	<0.001	
30-39	22.1 (20.9 - 23.3)	30.8 (29.5 - 32.0)	39.3 (38.1 - 40.6)	42.4 (40.7 - 44.1)	20.30 (18.23 - 22.36)	6.00 (3.27 - 8.81)	<0.001	
40-49	22.5 (21.2 - 23.8)	28.7 (27.3 - 30.1)	37.9 (36.4 - 39.3)	43.4 (41.4 - 45.4)	20.90 (18.52 - 23.28)	6.21 (4.66 - 7.78)	<0.001	
Residence								0.057
Urban	28.2 (27.0 - 29.4)	35.8 (34.6 - 37.0)	42.2 (41.1 - 43.4)	45.1 (43.4 - 46.8)	16.87 (14.81 - 18.94)	4.26 (2.40 - 6.15)	<0.001	
Rural	12.0 (11.4 - 12.6)	18.5 (17.8 - 19.2)	27.0 (26.2 - 27.8)	32.0 (30.8 - 33.1)	19.94 (18.61 - 21.26)	9.22 (6.07 - 12.47)	<0.001	
Educational attainment								0.027
No formal education	10.6 (9.6 - 11.6)	16.3 (15.3 - 17.3)	22.7 (21.5 - 24.0)	27.0 (24.8 - 29.2)	16.35 (13.92 - 18.78)	8.58 (4.59 - 12.71)	<0.001	
Primary	14.1 (13.1 - 15.1)	21.0 (20.0 - 22.0)	27.1 (26.1 - 28.2)	30.1 (28.5 - 31.6)	16.04 (13.75 - 18.33)	6.76 (3.61 - 10.02)	<0.001	
Secondary	20.5 (19.2 - 21.7)	27.5 (26.2 - 28.8)	36.7 (35.4 - 38.0)	39.3 (37.6 - 40.9)	18.79 (16.25 - 21.34)	6.19 (2.68 - 9.84)	<0.001	
Higher	31.5 (29.7 - 33.3)	37.6 (35.8 - 39.4)	43.1 (41.3 - 44.9)	47.3 (45.0 - 49.6)	15.81 (12.70 - 18.93)	3.96 (1.49 - 6.53)	<0.001	
Wealth quintile								0.009
Poorest	4.9 (4.1 - 5.7)	9.2 (8.1 - 10.2)	16.2 (15.0 - 17.4)	21.4 (19.4 - 23.3)	16.46 (14.36 - 18.57)	14.23 (9.68 - 18.96)	<0.001	
Poorer	6.7 (5.8 - 7.6)	13.1 (11.9 - 14.3)	21.2 (19.9 - 22.6)	29.7 (27.6 - 31.9)	23.02 (20.70 - 25.33)	14.10 (9.70 - 18.68)	<0.001	
Middle	11.6 (10.5 - 12.7)	19.7 (18.4 - 21.1)	30.5 (29.0 - 32.0)	34.2 (32.0 - 36.3)	22.57 (20.16 - 24.99)	10.25 (5.58 - 15.12)	<0.001	
Richer	20.3 (19.0 - 21.7)	28.3 (26.8 - 29.8)	36.5 (35.0 - 38.0)	39.3 (37.1 - 41.5)	19.00 (16.02 - 21.99)	5.81 (2.53 - 9.23)	<0.001	
Richest	29.3 (27.7 - 30.9)	32.7 (31.1 - 34.3)	35.6 (34.1 - 37.1)	38.6 (36.5 - 40.8)	9.35 (5.82 - 12.89)	2.33 (−0.17 - 4.84)	0.066	

*p-value for changes in prevalence over time; **p-value for difference of changes in prevalence over time among sociodemographic groups.

### Trend in obesity

Overall, prevalence of obesity showed a significant increase over the same period, rising from 17.7% in 2011 to 36.6% in 2022 according to the Asian cut-off (Fig 1), with an APC of 6.8%. On the other hand, prevalence of obesity according to WHO cut-off among reproductive-age women increased from 3.2% in 2011 to 8.0% in 2022, corresponding to an APC of 8.9% (S1 Table).

Age-stratified trends were broadly similar, with the younger women experiencing the most rapid rise (APC 7.1%) and slightly lower APCs among older age groups (6.0 to 6.2%; p = 0.812). Although, prevalence of obesity was higher among urban women, rural women had a faster increase (APCs 9.2% vs 4.3%; p = 0.057). The highest increase in prevalence of obesity was among women with no formal education (APC 8.6%) and slower growth among those with higher education (APC 4.0%; p = 0.027). The poorest and poorer quintiles had the largest APCs (14.2% and 14.1%), whereas the richest quintile showed minimal change (APC 2.3%; p = 0.009) (Table 2). These gradients persist when the obesity was defined according to the WHO cut-off, except for the wealth quintiles (S1 Table). Prevalence of obesity increased across all geographic regions, with the highest APC in Rangpur and Barisal divisions (10.9% and 9.2%, respectively). The urban-rural difference was highest in Barisal division, followed by Dhaka, Rangpur, and Sylhet divisions (S2 Table).

### Socioeconomic inequalities

**Fig 2:**
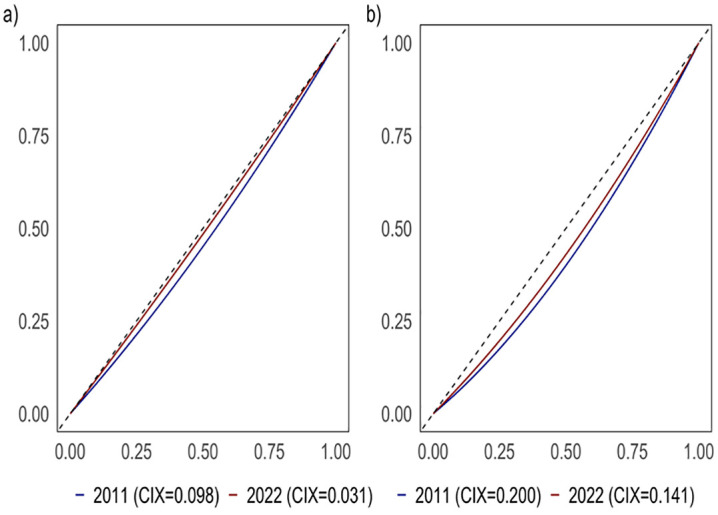
Concentration curves for prevalence of overweight and obesity among reproductive-age women with Erreygers-corrected concentration indices.

Prevalence of overweight and obesity among reproductive-age women in remained disproportionately concentrated among wealthier groups. Concentration curves for both indicators consistently lay below the line of equality, indicating a pro-rich distribution. However, the CIX for overweight declined from 0.098 in 2011 to 0.031 in 2022, while for obesity it declined from 0.200 to 0.141, suggesting a gradual trend toward a more equitable distribution across wealth quintiles, although disparities persist (Fig 2).

## Discussion

We observed a steep rise in prevalence of overweight and obesity among reproductive-age women in Bangladesh over the last decade. Urban women had higher baseline prevalence for both overweight and obesity, yet rural women experienced more rapid increases over time. Women with higher education showed the steepest increase in overweight, whereas those with no formal education experienced highest increase in prevalence of obesity. Socioeconomic inequalities persisted, with women from wealthier households maintaining the highest prevalence, although the fastest rates of increase were observed among women from lower-income groups.

Our findings are consistent with previous studies from Bangladesh [7,11,12], as well as other LMICs in Southeast Asia, such as India [23], Nepal [24], and Sri Lanka [25], which also similarly observed rising trends in overweight and obesity, particularly among women of reproductive age. However, the accelerated increase among women from lower-income groups in Bangladesh was higher compared to neighboring countries such as India and Nepal [23,26] which suggests a faster diffusion of obesogenic environments across socioeconomic strata in this country.

We observed an age-specific trend in prevalence of overweight and obesity among our participants. Women from older age groups continued to bear the highest absolute burden of overweight and obesity, however, younger women were experiencing a faster relative increase in prevalence of overweight. This phenomenon, although not statistically significant, has implication in perspective of reproductive health. Overweight and obesity in women of reproductive age are associated with adverse maternal and fetal outcomes, including gestational diabetes, pre-eclampsia, stillbirth, and cesarean delivery, as well as long-term risks for offspring such as childhood obesity and metabolic disorders [8–10]. This rising prevalence of overweight among younger women indicates that the population entering the childbearing age is increasingly at risk, suggesting that interventions addressing nutrition, physical activity, and weight management should start early, ideally before conception.

The rural-urban differences in prevalence of overweight and obesity observed in our study are particularly noteworthy. Urban women are consistently reported to have higher prevalence of overweight and obesity, which is consistent with the findings of previous studies from Bangladesh [13–16]. However, beyond observations of these studies, which primarily described rural-urban differences in prevalence of overweight and obesity, our analysis additionally evaluated differences in the rate of change in prevalence over time. We observed that rural women experienced a higher rate of increase in the prevalence of obesity compared to urban women. This may be driven by multiple factors, including rural exposure to processed foods, motorized transport, mechanized agriculture, and reduced physical labor [27]. Such shifts indicate the need to extend public health interventions traditionally focused on urban populations into rural areas, tailoring strategies to local contexts. Policy measures promoting healthier diets, community-based physical activity programs, and education on nutrition and weight management should be adapted for rural settings where awareness and resources may be limited.

Women from wealthier households continued to have higher prevalence of both overweight and obesity, as found in previous studies [13–16]. However, we observed the largest annual increases in the poorest and poorer quintiles, reflecting a rapid catch-up in overweight and obesity among lower-income women. A similar pattern was observed in the inequality analysis. Although the prevalence of overweight and obesity remained disproportionately higher among wealthier groups, indicating a persistent pro-rich distribution in CnI, the magnitude of inequality decreased over time. This pattern suggests that while overall inequality remains, the distribution is gradually becoming more equitable as the obesity burden shifts toward lower-income groups that historically had lower prevalence. Rising obesity rates in disadvantaged groups may exacerbate existing health inequities, as these populations often have less access to health services, preventive care, and education about healthy lifestyle practices. Furthermore, the intersection of gender norms, limited mobility, systemic barriers, and cultural expectations in Bangladesh places women at greater risk of sedentary behavior and restricted opportunities for physical activity [28,29].

Educational attainment also showed a similar gradient, as women with no formal education showing the steepest increase in prevalence of obesity. These findings are consistent with neighboring countries from South-east Asian region [23,26], where rapid economic growth have been accompanied by a widening of the obesity epidemic beyond affluent, urban populations. Such convergence between socioeconomic groups indicates a significant juncture for public health policy. If left unaddressed, this shift of obesity burden toward poorer women could exacerbate existing inequities in the burden of NCDs, given that these groups already face structural barriers to healthcare access, health literacy, and preventive services. Moreover, as disadvantaged populations typically have fewer resources to adopt healthier dietary patterns or engage in physical activity, they may be disproportionately affected by the long-term health and economic consequences of obesity. Targeted interventions, including community-based nutrition education, affordable access to healthy foods, and integration of obesity prevention within primary health care, would be essential to curb the rising burden of overweight and obesity in disadvantaged groups and to prevent further widening of health inequalities.

We additionally conducted regional analyses to examine trends in overweight and obesity across administrative divisions of Bangladesh. Prevalence remained consistently higher in divisions such as Dhaka and Chittagong throughout the study period. However, the annual rate of increase was greater in Rangpur and Barishal, where larger urban-rural differentials were also observed. A previous study by Hossain et al. (2022) have similarly reported disparities by education and household wealth in these regions [14]. Historically, these divisions have been considered relatively socioeconomically disadvantaged within the country [30,31]. Despite this, the faster rise in overweight and obesity in these areas further evidenced a rapid expansion of obesogenic environments, likely driven by ongoing economic growth, urbanisation, and lifestyle transitions. These findings indicate that the burden of overweight and obesity is no longer confined to traditionally affluent regions but is increasingly shifting towards populations that previously had lower prevalence, thereby contributing to a narrowing of regional and socioeconomic gaps over time.

Although our primary analysis used Asian-specific BMI cut-offs to define overweight and obesity, we repeated the analyses using the WHO standard BMI classification to assess the robustness of the findings. Because the WHO thresholds are higher than the Asian cut-offs, the WHO definition of overweight approximately corresponds to the Asian definition of obesity; accordingly, the increasing trend in overweight based on WHO criteria closely paralleled the obesity trend observed using Asian cut-offs. As expected, the prevalence of obesity defined by WHO criteria was substantially lower than that obtained using Asian thresholds, although the overall pattern of increase over time remained similar with both classifications, indicating a consistent upward shift in BMI distribution in the population. This pattern may be better understood by examining BMI as a continuous variable and evaluating population-level shifts across the entire distribution rather than relying only on categorical cut-offs [15]. Such analyses are also currently being done by our research team in a separate ongoing project, in which we are examining shifts across the full BMI spectrum using a quantile regression approach to quantify temporal changes at different BMI quantiles. However, the present findings suggest that, although prevalence of overweight and obesity is increasing in the population, most of the shift is still occurring within the lower range of the obesity spectrum. Severe obesity, as defined by BMI ≥ 30 kg/m², remains relatively less prevalent, indicating that the epidemic is still at an early stage and may provide an important window for timely prevention strategies.

The rising prevalence of overweight and obesity among women of reproductive age suggests that there is an urgent need to integrate obesity prevention into the national health agenda of Bangladesh. Existing maternal and reproductive health services offer an opportunity to deliver routine counselling on balanced diets, physical activity, and healthy weight maintenance. Community-based interventions targeting rural and low-income populations, where the fastest increases were observed, should provide culturally appropriate lifestyle guidance through local health workers and community groups.

Structural and regulatory measures are equally important. Policies to limit marketing of calorie-dense foods, introduce taxation on sugar-sweetened beverages, and incentivize healthier food production can help reshape the food environment. Investments in planning and infrastructure to encourage physical activity, including safe public spaces and active transport, are also important. A coordinated, multi-sectoral approach involving health, education, agriculture, and transport sectors is essential to curb obesity, prevent widening health inequalities, and reduce the future burden of NCDs in Bangladesh.

Our study had several strengths. We utilized nationally representative data from four rounds of the BDHS over more than a decade, enabling the analysis of long-term trends. Some previous studies, like Chowdhury et al. (2018) [11], documented rising overweight and obesity in Bangladesh using data from BDHS 1999–2014, with significant socioeconomic gradients across rural and urban populations. However, in our study, by incorporating more recent surveys (2017−18 and 2022), we reported more recent shifts and contemporary patterns of overweight and obesity, quantifying subgroup-specific rates of increase. These findings would provide updated evidence with relevance for public health policy, enabling interventions to address the ongoing rise in overweight and obesity while considering evolving regional, socioeconomic, and urban-rural dynamics. Standardized measurement of weight and height ensured reliable BMI classification, and the use of Asian-specific cut-offs allowed for epidemiologically relevant assessment of overweight and obesity. Our stratified analyses by age, residence, education, and wealth quintiles provided a detailed understanding of both absolute and relative trends, supporting evidence-informed policy design.

However, several limitations of our study merit consideration. The cross-sectional nature of the surveys precludes causal inference regarding the drivers of rising overweight and obesity. BMI, although widely used, does not distinguish between fat and lean mass, nor does it capture fat distribution such as abdominal obesity, which may have differential health implications. Data on dietary intake, physical activity, and other behavioral risk factors were limited due to utilization of secondary data, constraining the ability to explore mechanisms underlying observed trends. Additionally, despite consistent survey methodology, small differences in sampling frames or non-response patterns across survey years could introduce minor bias in trend estimates.

## Conclusions

In conclusion, our study demonstrated an increasing trend in prevalence of overweight and obesity among reproductive-age women in Bangladesh, with persistent socioeconomic gradients that are gradually becoming more equitable due to faster relative increases in lower-income and rural populations. Hence, population-level preventive interventions should target both urban and rural settings and across all socioeconomic strata. Policy and programmatic efforts should address the challenges of rising obesity, ensuring a comprehensive approach to curb the growing burden of non-communicable diseases in Bangladesh.

## Supporting information

S1 Table Trends in overweight and obesity among the participants according to WHO cut-off (n = 60,921). (PDF)

S2 Table Trends in overweight and obesity among the participants in different divisions of Bangladesh (n = 60,921). (PDF)
