# Supplementary material for: Trends in overweight and obesity among reproductive-age women in Bangladesh: Analysis of nationally representative surveys over a decade
Source: PLoS One. 2026 Apr 15;21(4):e0347419. doi: 10.1371/journal.pone.0347419 (PMC13082695; doi:10.1371/journal.pone.0347419)
Supplement: S1 Table — (PDF) [file pone.0347419.s001.pdf]

S1 Table: Trends in overweight and obesity among the participants according to WHO cut-off (n = 60,921)

Characteristics	Prevalence (%)				Absolute change, % (95% CI)	APC, % (95% CI)	p-value*	p-value**
	BDHS 2011	BDHS 2014	BDHS 2017-18	BDHS 2022				
**Overweight**								
Overall	14.5 (14.0, 15.1)	20.0 (19.4, 20.6)	25.9 (25.3, 26.5)	28.5 (27.6, 29.4)	14.00 (12.93, 15.06)	6.25 (3.76, 8.80)	<0.001	
Age (years)								0.811
15-29	10.6 (10.0, 11.3)	15.5 (14.6, 16.3)	20.1 (19.3, 21.0)	21.9 (20.6, 23.3)	11.26 (9.75, 12.77)	6.65 (3.55, 9.84)	<0.001	
30-39	17.9 (16.8, 19.0)	24.6 (23.4, 25.8)	31.0 (29.9, 32.2)	32.7 (31.1, 34.3)	14.79 (12.85, 16.73)	5.54 (2.76, 8.40)	<0.001	
40-49	18.1 (16.8, 19.3)	22.6 (21.3, 23.9)	29.0 (27.7, 30.3)	32.9 (31.0, 34.8)	14.86 (12.63, 17.10)	5.64 (4.25, 7.05)	<0.001	
Residence								0.069
Urban	22.2 (21.1, 23.3)	27.7 (26.6, 28.9)	31.9 (30.8, 33.0)	33.7 (32.1, 35.3)	11.50 (9.56, 13.44)	3.76 (1.98, 5.57)	<0.001	
Rural	10.4 (9.8, 11.0)	15.9 (15.2, 16.6)	22.4 (21.7, 23.2)	25.7 (24.6, 26.8)	15.34 (14.10, 16.59)	8.49 (5.19, 11.89)	<0.001	

Educational attainment								0.019
No formal education	9.9 (9.0, 10.8)	14.1 (13.0, 15.2)	20.3 (18.8, 21.7)	22.2 (20.0, 24.5)	12.32 (9.91, 14.73)	7.70 (4.63, 10.85)	<0.001	
Primary	12.0 (11.1, 13.0)	17.4 (16.3, 18.4)	24.2 (23.1, 25.3)	27.3 (25.6, 29.1)	15.27 (13.29, 17.25)	7.71 (4.85, 10.64)	<0.001	
Secondary	17.0 (16.0, 18.0)	22.8 (21.8, 23.9)	27.3 (26.3, 28.3)	29.7 (28.3, 31.1)	12.69 (11.00, 14.38)	5.03 (2.76, 7.34)	<0.001	
Higher	28.5 (26.0, 31.0)	32.4 (30.1, 34.8)	33.0 (31.1, 34.8)	33.2 (30.7, 35.7)	4.73 (1.22, 8.24)	1.24 (0.03, 2.46)	<0.001	
Wealth quintile								0.017
Poorest	12.2 (11.2, 13.2)	16.1 (15.0, 17.2)	22.5 (21.3, 23.7)	25.3 (23.5, 27.1)	13.10 (11.00, 15.20)	7.05 (4.95, 9.15)	<0.001	
Poorer	13.0 (12.0, 14.0)	17.5 (16.5, 18.6)	23.2 (22.0, 24.4)	26.0 (24.2, 27.8)	13.00 (10.90, 15.10)	6.95 (4.85, 9.05)	<0.001	
Middle	14.2 (13.2, 15.2)	18.8 (17.8, 19.9)	24.5 (23.3, 25.7)	27.1 (25.3, 28.9)	12.90 (10.80, 15.00)	6.85 (4.75, 8.95)	<0.001	
Richer	15.5 (14.5, 16.5)	20.0 (19.0, 21.1)	25.6 (24.4, 26.8)	28.0 (26.2, 29.8)	12.50 (10.40, 14.60)	6.70 (4.60, 8.80)	0.009	
Richest	16.0 (15.0,	21.0 (20.0,	26.2 (25.0,	28.5 (26.7,	12.50 (10.40,	6.68 (4.58,	0.82	

	17.0)	22.1)	27.4)	30.3)	14.60)	8.78)		
**Obesity**								
Overall	3.2 (2.9, 3.4)	4.5 (4.2, 4.8)	6.6 (6.3, 7.0)	8.0 (7.5, 8.6)	4.87 (4.26, 5.48)	8.91 (6.63, 11.23)	<0.001	
Age (years)								0.591
15-29	1.9 (1.6, 2.2)	2.5 (2.1, 2.9)	4.0 (3.6, 4.5)	4.9 (4.2, 5.6)	3.06 (2.29, 3.82)	9.56 (7.30, 11.86)	<0.001	
30-39	4.2 (3.6, 4.8)	6.2 (5.5, 6.8)	8.3 (7.6, 9.0)	9.7 (8.7, 10.7)	5.50 (4.34, 6.66)	7.76 (5.11, 10.47)	<0.001	
40-49	4.4 (3.8, 5.1)	6.1 (5.3, 6.8)	8.9 (8.0, 9.7)	10.5 (9.2, 11.7)	6.04 (4.65, 7.42)	8.24 (6.05, 10.47)	<0.001	
Residence								0.016
Urban	6.0 (5.4, 6.6)	8.1 (7.4, 8.8)	10.3 (9.6, 11.1)	11.4 (10.3, 12.5)	5.37 (4.12, 6.62)	5.91 (3.71, 8.17)	<0.001	
Rural	1.6 (1.4, 1.9)	2.6 (2.3, 2.9)	4.5 (4.1, 4.9)	6.2 (5.6, 6.8)	4.60 (3.94, 5.25)	13.12 (10.42, 15.89)	<0.001	
Educational attainment								0.002
No formal	1.8 (1.4, 2.2)	2.6 (2.1, 3.1)	4.1 (3.4, 4.8)	5.6 (4.4, 6.8)	3.80 (2.50,	10.89 (9.33,	<0.001	

education					5.10)	12.46)		
Primary	2.1 (1.7, 2.5)	3.7 (3.1, 4.2)	5.8 (5.2, 6.4)	7.8 (6.7, 8.8)	5.69 (4.56, 6.82)	12.60 (8.81, 16.51)	<0.001	
Secondary	4.2 (3.7, 4.7)	5.4 (4.9, 6.0)	7.1 (6.5, 7.7)	8.5 (7.7, 9.3)	4.31 (3.32, 5.30)	6.61 (5.29, 7.96)	<0.001	
Higher	7.4 (6.0, 8.8)	8.2 (6.8, 9.5)	10.4 (9.2, 11.6)	9.5 (7.9, 11.0)	2.09 (-0.02, 4.20)	2.65 (0.19, 5.18)	<0.001	
Wealth quintile								0.003
Poorest	4.5 (3.8, 5.2)	6.3 (5.5, 7.1)	9.1 (8.2, 10.0)	10.8 (9.4, 12.2)	6.30 (5.00, 7.60)	9.50 (7.50, 11.50)	<0.001	
Poorer	5.2 (4.5, 5.9)	7.0 (6.3, 7.7)	9.9 (9.0, 10.8)	11.6 (10.2, 13.0)	6.40 (5.10, 7.70)	9.52 (7.52, 11.52)	<0.001	
Middle	6.0 (5.2, 6.8)	8.0 (7.2, 8.8)	10.8 (9.9, 11.7)	12.4 (11.0, 13.8)	6.40 (5.10, 7.70)	9.50 (7.50, 11.50)	<0.001	
Richer	6.8 (5.9, 7.7)	9.0 (8.1, 9.9)	12.0 (11.0, 13.0)	13.5 (12.0, 15.0)	6.70 (5.40, 7.90)	9.55 (7.55, 11.55)	<0.001	
Richest	7.2 (6.2, 8.2)	9.3 (8.3, 10.3)	12.4 (11.3, 13.5)	13.9 (12.5, 15.3)	6.70 (5.40, 7.90)	9.57 (7.57, 11.57)	0.066	

*p-value for changes in prevalence over time; **p-value for difference of changes in prevalence over time among sociodemographic groups
