# Supplementary material for: Trends in overweight and obesity among reproductive-age women in Bangladesh: Analysis of nationally representative surveys over a decade
Source: PLoS One. 2026 Apr 15;21(4):e0347419. doi: 10.1371/journal.pone.0347419 (PMC13082695; doi:10.1371/journal.pone.0347419)
Supplement: S2 Table — (PDF) [file pone.0347419.s002.pdf]

S2 Table: Trends in overweight and obesity among the participants in different divisions of Bangladesh (n = 60,921)

Characteristics	Prevalence (%)				Absolute change, % (95% CI)	APC, % (95% CI)	p-value*	p-value**
	BDHS 2011	BDHS 2014	BDHS 2017-18	BDHS 2022				
**Overweight**								
Barisal								
Overall	13.0 (11.5-14.6)	14.7 (13.2-16.3)	17.7 (16.0-19.4)	18.3 (15.9-20.7)	5.23 (2.37, 8.08)	3.25 (1.99, 4.52)	<0.001	
Urban	18.4 (15.2-21.5)	18.8 (15.8-21.8)	18.3 (15.3-21.2)	19.0 (14.8-23.3)	0.68 (-4.57, 5.93)	0.22 (-0.27, 0.70)	0.382	0.015
Rural	10.4 (8.7-12.2)	12.8 (11.0-14.6)	17.4 (15.4-19.4)	17.9 (15.0-20.8)	7.42 (4.04, 10.8)	5.25 (2.88, 7.69)	<0.001	
Chittagong								
Overall	14.6 (13.2-16.0)	15.6 (14.2-17.0)	17.9 (16.5-19.4)	19.5 (17.4-21.6)	4.87 (2.37, 7.37)	2.76 (2.33, 3.19)	<0.001	
Urban	15.5 (13.2-17.8)	16.1 (13.8-18.5)	16.9 (14.5-19.2)	17.6 (14.5-20.7)	2.14 (-1.71, 5.99)	1.17 (1.08, 1.26)	<0.001	0.001
Rural	14.1 (12.4-15.8)	15.3 (13.6-17.0)	18.6 (16.7-20.4)	20.8 (18.0-23.7)	6.75 (3.45, 10.05)	3.79 (3.17, 4.40)	<0.001	

Dhaka								
Overall	12.8 (11.5-14.0)	16.7 (15.4-18.1)	18.0 (16.6-19.5)	19.0 (16.9-21.0)	6.21 (3.81, 8.61)	3.36 (1.06, 5.72)	0.004	
Urban	17.3 (15.2-19.4)	17.9 (15.8-20.0)	16.9 (15.0-18.7)	18.6 (15.7-21.6)	1.34 (-2.28, 4.97)	0.45 (-0.63, 1.54)	0.416	0.097
Rural	9.3 (7.8-10.7)	15.8 (14.1-17.6)	19.7 (17.4-22.0)	19.3 (16.4-22.2)	10.04 (6.82, 13.25)	6.51 (1.11, 12.19)	0.018	
Khulna								
Overall	14.6 (13.2-16.0)	17.0 (15.5-18.5)	17.8 (16.3-19.3)	19.1 (16.9-21.3)	4.51 (1.88, 7.13)	2.28 (1.22, 3.36)	<0.001	
Urban	14.4 (12.0-16.7)	18.3 (15.7-21.0)	16.5 (14.1-18.9)	19.4 (15.4-23.3)	5.02 (0.42, 9.62)	2.09 (-0.47, 4.71)	0.111	0.832
Rural	14.7 (13.0-16.4)	16.3 (14.5-18.1)	18.5 (16.6-20.5)	19.0 (16.3-21.7)	4.25 (1.04, 7.45)	2.40 (1.44, 3.38)	<0.001	
Rajshahi								
Overall	14.0 (12.6-15.4)	16.0 (14.5-17.4)	14.8 (13.3-16.4)	16.5 (14.2-18.8)	2.52 (-0.15, 5.19)	1.11 (-0.41, 2.66)	0.154	
Urban	16.0 (13.4-18.5)	15.5 (13.0-18.0)	15.7 (12.5-18.8)	16.4 (11.6-21.1)	0.42 (-4.97, 5.82)	0.27 (-0.34, 0.90)	0.385	0.355
Rural	13.0 (11.4-	16.2 (14.4-	14.6 (12.8-	16.5 (13.9-	3.54 (0.46, 6.62)	1.59 (-0.78,	0.191	

	14.6)	18.0)	16.3)	19.1)		4.02)		
Rangpur								
Overall	11.6 (10.3-12.9)	13.6 (12.3-15.0)	16.6 (15.1-18.0)	19.7 (17.5-22.0)	8.12 (5.53, 10.7)	4.92 (4.63, 5.22)	<0.001	
Urban	16.1 (13.3-18.9)	15.6 (12.9-18.3)	17.0 (14.4-19.6)	20.1 (16.3-23.9)	3.97 (-0.72, 8.66)	2.11 (0.60, 3.65)	0.006	0.016
Rural	9.8 (8.3-11.2)	12.9 (11.3-14.4)	16.3 (14.5-18.1)	19.5 (16.8-22.3)	9.77 (6.65, 12.88)	6.40 (5.01, 7.81)	<0.001	
Sylhet								
Overall	10.5 (9.1-11.9)	12.1 (10.6-13.5)	16.5 (15.0-18.0)	19.5 (17.2-21.8)	9.05 (6.36, 11.73)	6.08 (5.02, 7.15)	<0.001	
Urban	16.1 (13.3-18.9)	15.6 (12.9-18.3)	17.0 (14.4-19.6)	20.1 (16.3-23.9)	3.97 (-0.72, 8.66)	2.11 (0.60, 3.65)	0.006	0.016
Rural	8.9 (7.5-10.2)	12.6 (11.0-14.2)	26.9 (24.8-29.1)	34.9 (31.6-38.2)	26.07 (22.47, 29.66)	14.05 (10.18, 18.04)	<0.001	
**Obesity**								
Barisal								
Overall	15.5 (13.8-17.2)	20.9 (19.1-22.7)	33.1 (31.0-35.1)	39.7 (36.7-42.8)	24.22 (20.75, 27.68)	9.21 (6.89, 11.59)	<0.001	

Urban	27.4 (23.9-31.0)	36.6 (32.9-40.4)	41.0 (37.2-44.7)	48.9 (43.6-54.3)	21.50 (15.03, 27.97)	5.04 (3.24, 6.87)	<0.001	0.029
Rural	9.7 (8.1-11.4)	13.5 (11.7-15.3)	29.3 (26.9-31.7)	35.1 (31.5-38.8)	25.42 (21.43, 29.41)	13.31 (8.73, 18.07)	<0.001	
Chittagong								
Overall	19.1 (17.6-20.6)	28.3 (26.6-30.0)	39.6 (37.7-41.5)	41.4 (38.8-44.0)	22.32 (19.31, 25.34)	7.30 (3.56, 11.17)	<0.001	
Urban	25.2 (22.4-27.9)	38.2 (35.1-41.3)	46.3 (43.2-49.4)	45.3 (41.2-49.3)	20.07 (15.18, 24.96)	5.22 (0.97, 9.64)	0.015	0.286
Rural	15.5 (13.8-17.3)	22.7 (20.8-24.7)	35.8 (33.5-38.0)	38.6 (35.2-42.0)	23.08 (19.26, 26.91)	8.86 (5.09, 12.76)	<0.001	
Dhaka								
Overall	18.9 (17.4-20.3)	27.9 (26.3-29.6)	38.6 (36.7-40.4)	39.6 (37.0-42.2)	20.72 (17.77, 23.67)	6.96 (3.11, 10.96)	<0.001	
Urban	31.1 (28.5-33.7)	37.0 (34.3-39.6)	45.0 (42.5-47.4)	45.7 (42.0-49.5)	14.64 (10.08, 19.21)	3.67 (1.88, 5.49)	<0.001	0.101
Rural	9.5 (8.0-10.9)	21.0 (19.0-23.0)	29.5 (26.8-32.1)	33.9 (30.4-37.3)	24.43 (20.68, 28.18)	11.58 (4.49, 19.14)	0.001	
Khulna								
Overall	21.6 (20.0-	29.1 (27.4-	36.9 (35.0-	41.7 (38.9-	20.12 (16.89, 23.36)	6.07 (3.99,	<0.00	

	23.2)	30.9)	38.8)	44.5)		8.19)	1	
Urban	31.7 (28.6-34.8)	36.3 (33.1-39.6)	45.7 (42.5-48.9)	50.8 (45.8-55.8)	19.10 (13.21, 24.99)	4.53 (3.59, 5.48)	<0.001	0.165
Rural	16.1 (14.3-17.9)	25.4 (23.3-27.5)	31.8 (29.5-34.1)	37.4 (34.1-40.8)	21.30 (17.52, 25.08)	7.55 (4.20, 11.01)	<0.001	
Rajshahi								
Overall	18.4 (16.9-20.0)	25.4 (23.6-27.1)	24.4 (22.5-26.3)	26.3 (23.6-29.0)	7.92 (4.80, 11.04)	2.70 (-0.29, 5.78)	0.077	
Urban	28.8 (25.6-31.9)	37.4 (34.1-40.8)	38.2 (33.9-42.4)	36.2 (30.0-42.4)	7.44 (0.50, 14.38)	1.80 (-1.09, 4.77)	0.224	0.256
Rural	13.2 (11.6-14.9)	19.3 (17.3-21.2)	19.7 (17.7-21.7)	23.4 (20.4-26.4)	10.16 (6.77, 13.55)	4.65 (1.65, 7.74)	0.002	
Rangpur								
Overall	12.6 (11.2-13.9)	19.0 (17.4-20.5)	32.1 (30.3-34.0)	38.5 (35.8-41.2)	25.92 (22.88, 28.96)	10.96 (7.57, 14.46)	<0.001	
Urban	21.6 (18.5-24.8)	34.7 (31.1-38.3)	42.5 (39.1-45.9)	45.0 (40.3-49.7)	23.39 (17.76, 29.02)	6.51 (2.27, 10.92)	0.002	0.066
Rural	8.9 (7.5-10.2)	12.6 (11.0-14.2)	26.9 (24.8-29.1)	34.9 (31.6-38.2)	26.07 (22.47, 29.66)	14.05 (10.18, 18.04)	<0.001	
Sylhet								

Overall	16.3 (14.6-18.0)	17.4 (15.7-19.2)	26.9 (25.1-28.7)	32.0 (29.3-34.7)	15.68 (12.50, 18.86)	6.92 (4.67, 9.22)	<0.001	
Urban	21.6 (18.5-24.8)	34.7 (31.1-38.3)	42.5 (39.1-45.9)	45.0 (40.3-49.7)	23.39 (17.76, 29.02)	6.51 (2.27, 10.92)	0.002	0.066
Rural	8.9 (7.5-10.2)	12.6 (11.0-14.2)	26.9 (24.8-29.1)	34.9 (31.6-38.2)	26.07 (22.47, 29.66)	14.05 (10.18, 18.04)	<0.001	

*p-value for changes in prevalence over time; **p-value for difference of changes in prevalence over time among sociodemographic groups
